# Editorial: The crosstalk between metabolism and inflammation in aging and longevity

**DOI:** 10.3389/fendo.2025.1734527

**Published:** 2025-12-15

**Authors:** Sandeep Kumar, Ahmed Elmansi, Mohamed Noureldein, James Harper

**Affiliations:** 1 Independent Researcher, New Orleans, LA, United States; 2 Department of Internal Medicine, Division of Cardiovascular Medicine, University of Michigan, Ann Arbor, MI, United States; 3 Department of Neurology, University of Michigan, Ann Arbor, MI, United States; 4 Department of Biological Sciences, Sam Houston State University, Huntsville, TX, United States

**Keywords:** aging, senescence, inflammation, mitochondrial stress, cell death, metabolism, longevity

## Introduction

Aging is a complex and multifaceted process involving a variety of interrelated molecular mechanisms and cellular systems. It is characterized by continuous deterioration in physiological functions across nearly all cells and tissues (1–4). During the lifespan, two main processes increasingly emerge as key problems: disturbance in metabolism and low‐grade chronic inflammation, also known as inflammaging. One of the hallmarks of aging is the decline in mitochondrial function leading to excess generation of reactive oxygen species (ROS), impaired energy production, and metabolic inflexibility (5, 6). These mitochondrial defects are sensed by immune cells (e.g. macrophages, monocytes), which may shift toward glycolytic metabolism over oxidative phosphorylation or fatty acid oxidation. These shifts, observed in aged immune cells, are associated with upregulation of inflammatory pathways (e.g. NF-κB, NLRP3 inflammasome). On the other hand, there is a loss of function in nutrient‐sensing pathways (mTOR, AMPK). NAD^+^ levels decline with age, and therefore, sirtuin activity drops, leading to impaired regulatory control over inflammation and deficient repair responses (7).

This Research Topic aimed to explore and advance our understanding of the complex interplay between metabolic processes and inflammatory responses, specifically, how these biological phenomena pertain to aging and lifespan extension and how these processes mutually influence one another. Supplementation or enhancement of NAD^+^ biosynthesis has thus gained attention as a strategy to re-balance metabolism and restrain inflammation (8). Dietary approaches such as caloric restriction (CR), intermittent fasting, and CR mimetics have repeatedly demonstrated efficacy in animal models for reducing inflammation and improving metabolic markers; human clinical evidence is growing. Pharmacological agents like metformin, rapamycin, and small molecules affecting metabolic regulators (e.g. AMPK activators, NAD^+^ precursors) show promise in preclinical models (9). Another interesting therapeutic target is the gut microbiota, particularly microbial metabolism of dietary components such as tryptophan, which appears to influence systemic inflammation, frailty, and muscle decline in aging (10). In total, seven studies were published under this Research Topic. These are summarized below.

## Highlights of the Research Topic

Choi et al.’s study aimed to assess the inflammatory metabolic activity of visceral adipose tissue (VAT) using 18F-FDG PET/CT to investigate its association with retinal vein occlusion (RVO) in elderly individuals. They concluded that metabolic activity of VAT, as assessed by 18F-FDG PET/CT, was associated with the presence of RVO and correlated with the degree of systemic inflammation. Therefore, VAT maximum standardized uptake value (SUVmax) may serve as a potential surrogate marker for obesity-related VAT inflammation linked to RVO.

James et al. reported that the membrane transporter progressive ankylosis protein homologue *ANKH/SLC62A1* (ANKH) and extracellular citrate (EC) are upregulated concomitantly in a variety of senescent cells and that both are regulated by some of the molecular pathways that regulate senescence-associated secretory phenotype (SASP) proteins, but not all. These findings identify a potentially important role of ANKH/Ank in the regulation of EC in many types of senescent cells and, by inference, some age-related diseases. In conclusion, this study finds that the novel citrate plasma membrane exporter ANKH is upregulated in various senescent cell types and is regulated by established drug targets. This finding suggests new strategies for countering the deleterious effects of senescent cells and telomere attrition. However, the authors note that ANKH and citrate may have opposing effects in humans and mice as well as in different ageing tissues, depending on dietary factors.

Yang et al. investigated the relationship between Weight Adjusted Weight Index (WWI), Systemic Immune-Inflammation Index (SII), and sKlotho in people aged over 45 in the United States using a cross-sectional study based on National Health and Nutrition Examination Survey (NHANES) data to determine whether SII plays a mediating role in the relationship between WWI and sKlotho. Using multivariate linear regression, they found a negative correlation between WWI and sKlotho, and that SII may be an important mediator between WWI and sKlotho. They further hypothesized that reducing inflammatory conditions in obese populations may increase sKlotho level, which in turn may delay organismal aging in middle-aged and elderly people.

Zhao and Liang aimed to investigate the association between the Triglyceride-Glucose Index (TyG) and PhenoAgeAccel, a measure of the difference between one’s PhenotypicAge and ChronologicalAge, using NHANES data. They examined threshold effects and stratified relationships to provide new insights into metabolic drivers associated with biological aging, and to inform potential intervention strategies for mitigating age-related health risks. They concluded that a higher TyG index level, particularly above a threshold of 9.60, is significantly associated with accelerated biological aging. These findings suggest the importance of metabolic health in biological aging processes and potential interventional strategies.

Yan et al. investigated the possible role of Klotho in longevity and disease prevention. Previous studies have shown that inflammation can reduce Klotho expression, leading to speculation that anti-inflammatory drugs may help maintain or even increase Klotho levels. However, they observed that the use of Non-steroidal Anti-Inflammatory Drugs (NSAIDs) is associated with a decrease in serum Klotho levels.

Shu et al. aimed to investigate the relationship between metabolically healthy individuals across multiple BMI categories and serum Klotho levels using NHANES data, which provides comprehensive information on serum Klotho levels. This large population-based study concluded that Klotho levels vary according to metabolically healthy status across regardless of BMI category, with metabolically unhealthy phenotypes exhibiting notably lower levels. These findings highlight the influence of metabolic abnormalities and body fatness on the aging process.

Li et al. provided a comprehensive perspective of the mechanisms underlying cellular senescence, a critical biological process with both beneficial and detrimental implications that is dependent on context. Specifically, while senescence is essential for tissue repair, embryogenesis, and tumor suppression by halting the proliferation of damaged cells, the accumulation of senescent cells and their senescence-associated secretory phenotype (SASP) contributes to chronic inflammation, tissue dysfunction, and the progression of age-related diseases such as cancer, diabetes, and neurodegenerative disorders. This timely review highlights the biological significance of cellular senescence and offers new insights for the development of anti-senescence strategies.

## Molecular and cellular mechanisms involved in aging and longevity

**Figure 1:**
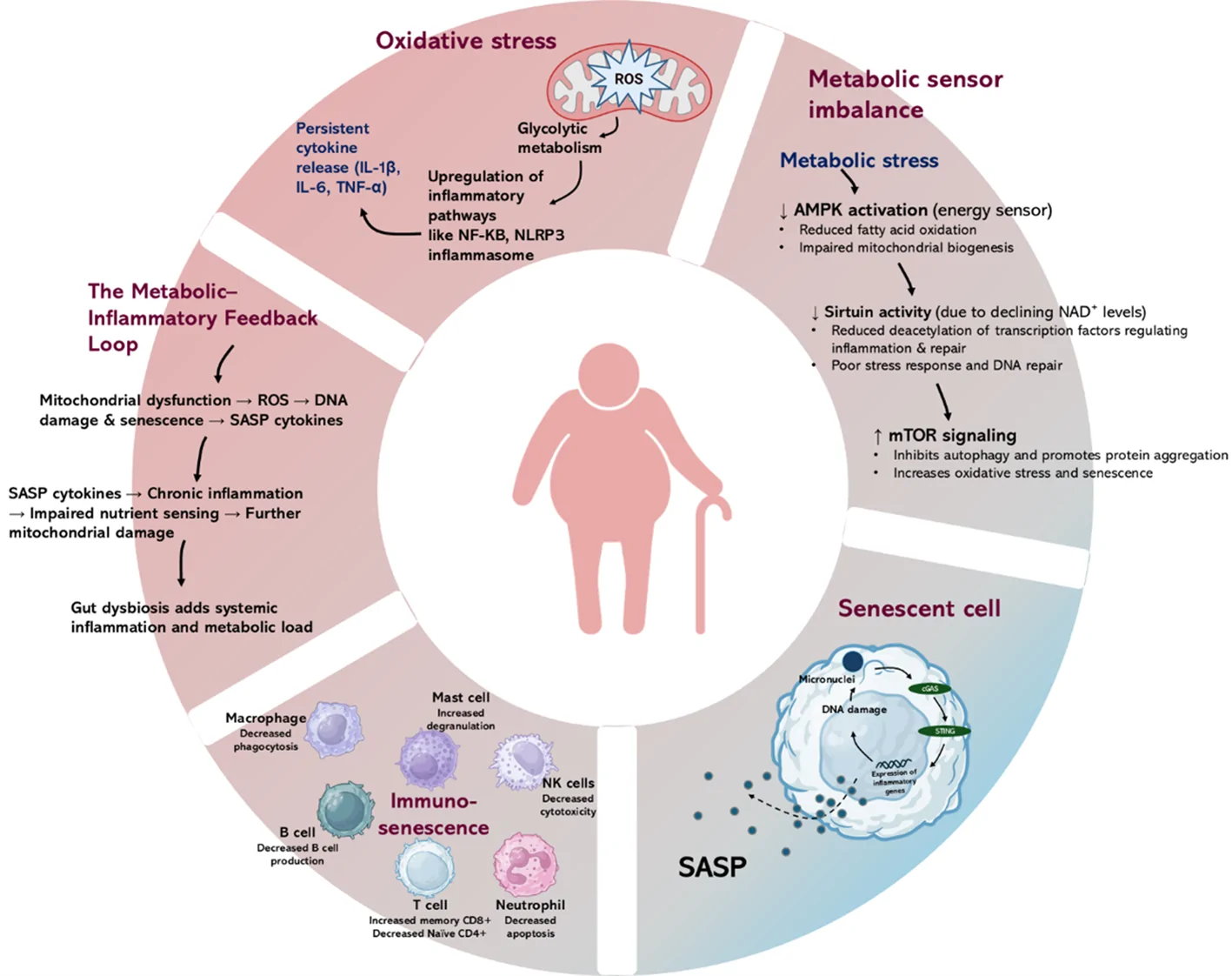
Representation of molecular, cellular, metabolic and Immune mechanisms in aging.

Organismal aging is due in part to oxidative stress subsequent to the generation of reactive oxygen species (ROS), mitochondrial dysfunction, and impaired antioxidant defense systems which disturb metabolic homeostasis, DNA repair and cellular health (11). Mitochondrial and cellular energy systems, exacerbating ROS production and igniting inflammatory signaling trigger cellular senescence, a state of growth arrest plus a pro-inflammatory secretory phenotype (SASP) which not only impairs tissue renewal but fosters local and systemic inflammation (12). Moreover, the aging immune system suffers immune-senescence, diminished capacity of adaptive and innate responses, altered cell subsets, and increased low-grade chronic inflammation. Increasing longevity and health span will likely result from interrupting the vicious cycles connecting metabolic stress, inflammation and senescence. Metabolic impairment promotes inflammation (via senescent and immune cells), and inflammation in turn exacerbates metabolic dysfunction and cellular damage (13). Mitochondrial dysfunction plays a central role: with age, accumulation of mtDNA damage, reactive oxygen species (ROS) production and impaired nutrient-sensing (e.g., NAD^+^ decline, AMPK/mTOR dysregulation) disrupts metabolic homeostasis and generates metabolic stress. This metabolic stress feeds into inflammation via activation of key signaling pathways: for example, ROS and mitochondrial DAMPs (damage-associated molecular patterns) trigger the NF-κB signaling pathway, the cGAS-STING pathway, and toll-like receptor and inflammasome responses (11, 14). The activated inflammatory responses contribute to the induction of cellular senescence: DNA damage responses (DDR), telomere shortening, epigenetic changes and metabolic imbalance promote the expression of cell-cycle inhibitors such as p16 p21and arrest the cell proliferation (15). Senescent cells adopt the senescence-associated secretory phenotype (SASP), releasing pro-inflammatory cytokines (IL-6, IL1 β, TNF-α), chemokines, extracellular vesicles and DAMPs, which further amplify inflammation and dysregulate neighboring cells and tissues. Immuno-senescence develops ageing immune cells (especially T cells, and macrophages), mitochondrial dysfunction and reduced lysosomal/autophagic clearance. Nutrient-sensing and metabolic regulators, the mTOR signaling pathway and AMP-activated protein kinase (AMPK) regulate metabolic responses to nutrient status; dysregulation of these with age impairs metabolic flexibility and increases stress. Sirtuins and NAD^+^ metabolism: decline of NAD^+^ affects sirtuin activity, impairing mitochondrial maintenance, causing ROS accumulation and promoting inflammation/senescence (16, 17) (**Figure 1**).

## Gap in knowledge

It is well known that metabolism and inflammation play important roles in the progression of cell and organ senescence and promote early aging, through major gaps remain in pinpointing causality, elucidating tissue and organ specific mechanistic pathways, translating these findings to clinical relevance in humans by optimizing intervention strategies, and accounting for the broader context such as environmental, genetical and lifestyle factors. Addressing these multiple gaps will be necessary to design effective interventions to promote healthy ageing and extend lifespan and healthspan.

## Future perspectives

The metabolic inflammatory axis plays a vital role in aging and longevity: disruptions in lipid and triglyceride metabolism promote mitochondrial dysfunction, oxidative stress and altered nutrient‐sensing, which in turn trigger cellular senescence and the pro-inflammatory senescence-associated secretory phenotype (SASP). Moreover, chronic low-grade inflammation again impairs metabolic homeostasis and creates a self-reinforcing loop that drives organ decline and shortens lifespan. Thus triglyceride/lipid metabolism dysregulation modulates inflammatory conditions and reprogramming of stages of senescent.

## Conclusion

This Research Topic offers a comprehensive overview of emerging metabolic and inflammatory pathways that contribute to diverse phenotypes of aging and longevity. The topic contributions addressed key regulatory pathways involved in modulating the progression of premature senescence and inflammation and discussed their potential implications for future therapeutic strategies. Enhancing metabolic homeostasis, dampening inflammation, and/or eliminating senescent cells hold real promise for extending lifespan and healthspan. However translation of these observations into improved human health and longevity requires deeper mechanistic understanding of biological aging at the cellular, tissue and organismal levels, development of specific biomarkers for various age related diseases and conditions and well defined intervention strategies.

## References

[1] Li Y Tian X Luo J Bao T Wang S Wu X . Molecular mechanisms of aging and anti-aging strategies. Cell Commun Signal. (2024) 22:285. doi: 10.1186/s12964-024-01663-1 38790068 PMC11118732

[2] Zhang K Kan C Luo Y Song H Tian Z Ding W . The promotion of active aging through older adult education in the context of population aging. Front Public Health. (2022) 10:998710. doi: 10.3389/fpubh.2022.998710 36299739 PMC9589353

[3] Ogrodnik M . Aging: the wound that never starts healing. Nat Commun. (2025) 16:8732. doi: 10.1038/s41467-025-64462-3 41027926 PMC12484887

[4] Zhu S Storlino G Kumar S . The endocrine role of the musculoskeletal system. Front Endocrinol. (2025) 16:1552950. doi: 10.3389/fendo.2025.1552950 39917542 PMC11798814

[5] Yang J Luo J Tian X Zhao Y Li Y Wu X . Progress in understanding oxidative stress, aging, and aging-related diseases. Antioxidants. (2024) 13:394. doi: 10.3390/antiox13040394 38671842 PMC11047596

[6] Zhao Q Liu J Deng H Ma R Liao JY Liang H . Targeting Mitochondria-located circRNA SCAR alleviates NASH via reducing mROS output. Cell. (2020) 183:76–e9322. doi: 10.1016/j.cell.2020.08.009 32931733

[7] Angajala A Lim S Phillips JB Kim JH Yates C You Z . Diverse roles of mitochondria in immune responses: novel insights into immuno-metabolism. Front Immunol. (2018) 9:1605. doi: 10.3389/fimmu.2018.01605 30050539 PMC6052888

[8] Covarrubias AJ Perrone R Grozio A Verdin E . NAD^+^ metabolism and its roles in cellular processes during ageing. Nat Rev Mol Cell Biol. (2021) 22:119–41. doi: 10.1038/s41580-020-00313-x 33353981 PMC7963035

[9] Du H Xu T Yu S Wu S Zhang J . Mitochondrial metabolism and cancer therapeutic innovation. Sig Transduct Target Ther. (2025) 10:245. doi: 10.1038/s41392-025-02311-x 40754534 PMC12319113

[10] Grosicki GJ Fielding RA Lustgarten MS . Gut microbiota contribute to age-related changes in skeletal muscle size, composition, and function: biological basis for a gut-muscle axis. Calcif Tissue Int. (2018) 102:433–42. doi: 10.1007/s00223-017-0345-5 29058056 PMC5858871

[11] Xu X Pang Y Fan X . Mitochondria in oxidative stress, inflammation and aging: from mechanisms to therapeutic advances. Signal Transduction Targeted Ther. (2025) 10:190. doi: 10.1038/s41392-025-02253-4 40500258 PMC12159213

[12] Stojanovic B Jovanovic I Dimitrijevic Stojanovic M Stojanovic BS Kovacevic V Radosavljevic I . Oxidative stress-driven cellular senescence: mechanistic crosstalk and therapeutic horizons. Antioxidants. (2025) 14:987. doi: 10.3390/antiox14080987 40867884 PMC12383077

[13] Zhang X Gao Y Zhang S Wang Y Pei X Chen Y . Mitochondrial dysfunction in the regulation of aging and aging-related diseases. Cell Communication Signaling. (2025) 23:290. doi: 10.1186/s12964-025-02308-7 40537801 PMC12177975

[14] Cai J Chen Z Yang X Wen Cai J Chen L Chen . Role of Mitochondrial damage-associated molecular patterns in osteoarthritis: from pathogenesis, diagnosis, and prognosis to therapeutics. Pharmacol Res. (2025) 219:107865. doi: 10.1016/j.phrs.2025.107865 40712761

[15] Shreeya T Ansari MS Kumar P Saifi M Shati AA Alfaifi MY . Senescence: A DNA damage response and its role in aging and neurodegenerative diseases. Front Aging. (2024) 4:1292053. doi: 10.3389/fragi.2023.1292053 38596783 PMC11002673

[16] Cuollo L Antonangeli F Santoni A Soriani A . The senescence-associated secretory phenotype (SASP) in the challenging future of cancer therapy and age-related diseases. Biology. (2020) 9:485. doi: 10.3390/biology9120485 33371508 PMC7767554

[17] Han Z Wang K Ding S Zhang M . Cross-talk of inflammation and cellular senescence: a new insight into the occurrence and progression of osteoarthritis. Bone Res. (2024) 12:69. doi: 10.1038/s41413-024-00375-z 39627227 PMC11615234

